# Rapid wall shear stress prediction for aortic aneurysms using deep learning: a fast alternative to CFD

**DOI:** 10.1007/s11517-025-03311-3

**Published:** 2025-02-17

**Authors:** Md. Ahasan Atick Faisal, Onur Mutlu, Sakib Mahmud, Anas Tahir, Muhammad E. H. Chowdhury, Faycal Bensaali, Abdulrahman Alnabti, Mehmet Metin Yavuz, Ayman El-Menyar, Hassan Al-Thani, Huseyin Cagatay Yalcin

**Affiliations:** 1 Biomedical Research Center, Qatar University, Doha, 2713 Qatar; 2 Department of Electrical Engineering, Qatar University, Doha, 2713 Qatar; 3 Electrical and Computer Engineering Department, The University of British Columbia, Vancouver, V6T 1Z4 BC Canada; 4 Heart Hospital, Hamad Medical Corporation, Doha, 3050 Qatar; 5 Department of Mechanical Engineering, Middle East Technical University, Ankara, 06800 Turkey; 6 Department of Surgery, Trauma, and Vascular Surgery, Hamad Medical Corporation, Doha, 3050 Qatar; 7 Clinical Medicine, Weill Cornell Medical College, Doha, 24144 Qatar; 8 Department of Biomedical Science, College of Health Sciences, QU Health, Qatar University, Doha, 2713 Qatar; 9 Department of Mechanical and Industrial Engineering, Qatar University, Doha, 2713 Qatar

**Keywords:** Abdominal aortic aneurysm, Deep learning, Neural network, Artificial intelligence, Hemodynamics, Computational fluid dynamics

## Abstract

**Abstract:**

Aortic aneurysms pose a significant risk of rupture. Previous research has shown that areas exposed to low wall shear stress (WSS) are more prone to rupture. Therefore, precise WSS determination on the aneurysm is crucial for rupture risk assessment. Computational fluid dynamics (CFD) is a powerful approach for WSS calculations, but they are computationally intensive, hindering time-sensitive clinical decision-making. In this study, we propose a deep learning (DL) surrogate, MultiViewUNet, to rapidly predict time-averaged WSS (TAWSS) distributions on abdominal aortic aneurysms (AAA). Our novel approach employs a domain transformation technique to translate complex aortic geometries into representations compatible with state-of-the-art neural networks. MultiViewUNet was trained on $\varvec{23}$ real and $\varvec{230}$ synthetic AAA geometries, demonstrating an average normalized mean absolute error (NMAE) of just $\varvec{0.362\%}$ in WSS prediction. This framework has the potential to streamline hemodynamic analysis in AAA and other clinical scenarios where fast and accurate stress quantification is essential.

**Graphical abstract:**

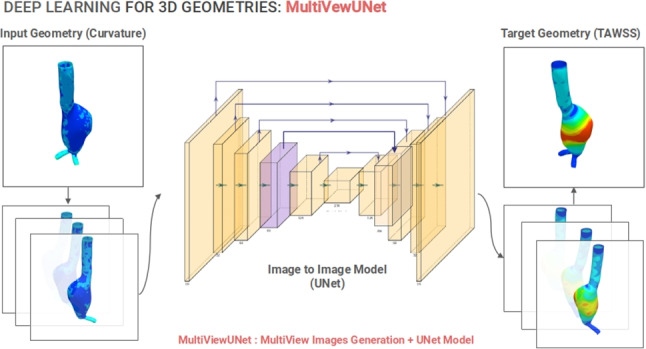

**Supplementary Information:**

The online version contains supplementary material available at 10.1007/s11517-025-03311-3.

## Introduction

In the last two decades, cardiovascular diseases (CVDs) have become the largest cause of non-communicable disease mortalities worldwide. The World Health Organization (WHO) estimates that 17.6 million people died of CVDs worldwide in 2012 alone, proportionally accounting for an estimated 31.3% of global mortality [1]. Among the CVDs, aortic aneurysms is one of the most prevalent and serious conditions where aortic tissue becomes degenerative and is formed into an abnormal balloon-shaped expansion. These aneurysms mainly affect the abdominal side, referred to as abdominal aortic aneurysms (AAA), and a patient is usually diagnosed with the condition when the aortic dilatation surpasses 50 percent of the normal vessel diameter [2]. According to the statistics, 4–8% of males and 0.5–1% of females over 50 have AAA, which is responsible for 15,000 deaths annually in the United States alone [3, 4]. These incidence rates highlight the vital need for early detection and treatment of AAAs.

The formation of AAA can be tied to the dynamic action of blood flow known as hemodynamics. Due to the hemodynamic effects of the blood flow, the luminal and endothelial wall surfaces of arteries are continuously exposed to frictional stress known as wall shear stress (WSS). Several investigations [5, 6] have revealed that shear stress directly affects vessel wall remodeling. The existence of an intraluminal thrombus (ILT) as well as the ballooning of the sac influence the hemodynamics of AAA. This flow disturbance led to the formation of circulatory flow zones with low WSS and high oscillation which were shown to contribute to the progression of AAA and eventual rupture [7–11]. Rupture is the worst AAA scenario with 80% mortality [11]. As low WSS and high oscillation regions within the aorta can be indicative of AAA and eventual rupture, examining disturbed AAA hemodynamics would be useful for rupture risk assessment [5, 12]. Several WSS-related hemodynamics parameters were defined to quantify disturbed hemodynamics within the cardiovascular system including the aorta, such as time-averaged WSS (TAWSS), oscillatory shear index (OSI), endothelial cell activation potential (ECAP), and relative residence time (RRT) [13]. Previously aortic aneurysms were shown to be prone to rupture in low TAWSS and high OSI, ECAP, and RRT regions [13]. Hence, precise calculation of these parameters might help to predict rupture prior to its clinical occurrence. In this study, we focus our attention on the TAWSS for characterizing aneurysms.

Computational fluid dynamics (CFD) is a powerful approach for the assessment of hemodynamics and calculation of relevant parameters such as WSS for investigating different cardiovascular diseases. In addition, several CFD-based diagnostic and decision assistance systems have been proposed [5, 14–17]. CFD techniques have also been extensively used to explore complicated flow patterns in the aorta [17–19], and a number of studies have attempted to correlate CFD hemodynamic parameters with the progression and rupture risk of AAA [5, 6, 20–24]. However, due to the intricacy of the underlying physical problem of viscous three-dimensional fluid flow, such procedures are extremely specialized, time-consuming, and require expertise in both engineering and medicine. Machine learning (ML) has been suggested as an approach to mitigate the limitations of CFD mentioned above. ML potentially can expedite CFD run times while keeping the accuracy of the simulations. Therefore, numerous promising applications of ML-based hemodynamic modeling have yet to be put into clinical practice.

In recent years, the enormous increase in computer power and data storage capacity has resulted in a paradigm shift in the deep learning (DL), allowing its broad usage in all medical research fields [25–27]. Not until recently, however, have DL algorithms been implemented in high-dimensional, complex dynamic systems, such as fluid dynamics [28–31]. As a result, numerous papers have attempted to analyze biological fluid flows in an effort to reduce the high simulation times of conventional CFD simulations. In this regard, Liang et al. [32], published in 2018, proposed a deep neural network (DNN)-based technique to directly estimate the WSS of the aorta without conducting finite element analysis (FEA). Based on their work, Morales et al. [33] created a DNN in 2019 to develop a fast surrogate of the left atrial appendage (LAA), rapidly assessing the risk of thrombus formation in the LAA without performing fluid simulations. Unfortunately, his work had some limitations, particularly in the pre-processing phase of the meshes, where training of the network was required. All LAAs required the same number of nodes with identical connectivity, achieved by registering each shape to a template. This process introduced coarse triangular elements needing manual smoothing and suffered from registration faults due to significant discrepancies. The LAAs had to be carefully built and aligned for correct orientation. Furthermore, the non-Cartesian registration limited the use of advanced neural network architectures like CNNs, which are highly effective in image processing tasks [34, 35].

In order to use the DL for CFD prediction, an appropriate approach needs to be adapted that can handle 3D shapes as inputs and outputs. 3D geometries are represented by a graph data structure that is not Euclidian and unsuitable for traditional neural network architecture. While 3D geometric CNN [36] and graph CNN [37] networks are addressing this issue, these networks are fairly new and are being investigated for optimization in the present days. Therefore, a realistic method of getting an Euclidean representation of the data is necessary so that it may be supplied to the traditional CNN architectures. Here, we proposed a new approach for representing 3D aorta geometries with a set of 2D images. Converting a 3D geometry into 2D images has several benefits. First, we get the benefit of applying CNN networks for analysis which are much more well-researched compared to 3D graphical neural networks. Another benefit is that the geometry does not have to be uniform or in other words, different geometries do not need to contain the same number of nodes. They can contain an arbitrary number of nodes in 3D, but the generated images in 2D will be of the same size. This limitation has been faced by similar works resulting in registration faults as discussed earlier. Lastly, our approach is not only applicable for this specific problem but it can also be generalized to other problem domains that deal with 3D geometries.

**Fig. 1:**
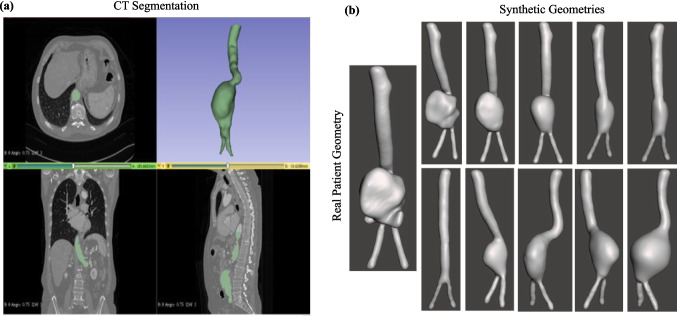
**a** 3D Geometry extraction from CT images. **b** 10 synthetic geometry generation from a single real patient’s geometry

In this study, to investigate aorta hemodynamics, a total of 253 aortic geometries were constructed containing 23 actual patient geometries and 230 synthetically generated geometries. After that, CFD simulations were run on each geometry to generate the TAWSS pattern on the aortic wall. The proposed MultiViewUNet framework was used to predict the TAWSS pattern of the aorta geometry and these results were then compared with the CFD results revealing comparable TAWSS profiles. To the best of our knowledge, this work is the first to explore WSS distribution for AAA using DL approach. The key contributions of this work can be summarized as follows.

- A real-time DL surrogate was proposed that bypasses the need for CFD simulation for predicting the WSS distribution on the aortic wall.
- A new domain transformation approach was proposed for geometry-to-geometry analysis, compatible with the existing state-of-the-art image-to-image networks.
- A novel neural network architecture called MultiViewUNet was proposed, which takes advantage of the domain transformation and enhances the capability of the vanilla UNet architecture.

## Methodology

The proposed methodology contains several steps discussed in the following subsections. First, we provide the details about our dataset and AAA geometry generation process. Next, the CFD analysis process has been reported. After that, we explain the features that were used for training the DL algorithm including geometry curvatures. Following that, the geometry-to-image conversion process has been discussed. Next, the data augmentation process has been described. Finally, we introduce our proposed network architecture, loss function, and evaluation metrics.

### Generation of synthetic geometries

The Hamad General Hospital’s Department of Surgery, Trauma, and Vascular Surgery Center provided CT scans of the aortas of 23 individuals that were diagnosed with AAA. Data collection was carried out under the IRB ethical approval obtained from Hamad Medical Corporation (MRC-02-20-134). There are 19 male patients and 4 female patients in the study population. Patients’ average ages were 62 for men and 69 for women, respectively. The male group’s youngest patient is 31 years old, while the female is 39. Due to the scarcity of patients’ AAA geometries for DL applications, we carried out an approach to increase the number of AAA geometries for enhancing the quality of DL training. For this purpose, 10 distinct synthetic geometries were generated manually for each of the 23 original AAA geometries. A total of 230 synthetic geometries were created with this method with the guidance of clinician experts of the team for possible similar AAA geometries in each case. Here, 3D Slicer (www.slicer.org) was used for CT image segmentation, whereas Mesh Mixer was used for modifying original geometries to create synthetic geometries (Autodesk Inc.). Figure 1a presents an example case where an original 3D AAA geometry is generated from patient CT scans and Fig. 1b shows the generation of 10 distinct synthetic geometries from the original AAA geometry.

### CFD analysis

Details of our CFD approach for AAA hemodynamics are explained in detail in our previous papers [5, 6, 13], hence it is briefly explained here.

**Fig. 2:**
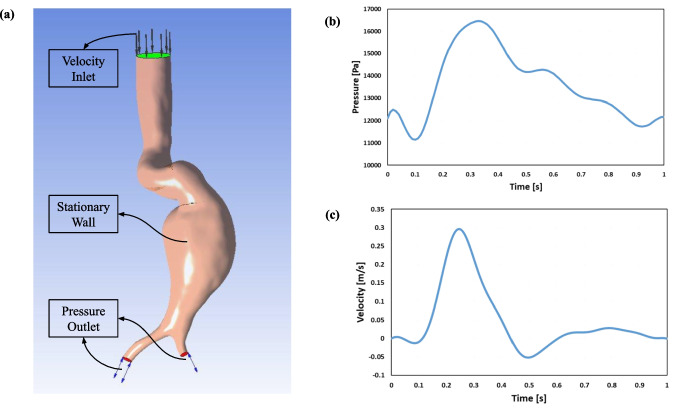
Transient CFD analysis boundary conditions. **a** Boundary condition locations on the AAA model. **b** Velocity-Time graph used as an inlet condition. **c** Pressure–time graph used as an outlet condition

**Numerical model:** In the CFD analysis, blood was assumed to be a homogeneous, incompressible fluid. The numerical model solves governing equations such as continuity (Eq. 1), modified momentum (Eq. 2), and turbulence model equations.

1 $$\begin{aligned} \frac{\partial \rho }{\partial t} + \frac{\partial }{\partial x_j}(\rho u_j) = 0 \end{aligned}$$

2 $$\begin{aligned} \frac{\partial }{\partial t}(\rho u_i) + \frac{\partial }{\partial x_j}(\rho u_i u_j) = \frac{\partial P}{\partial x_i} + \frac{\partial }{\partial x_j}[\mu _{eff}(\frac{\partial u_i}{\partial t} + \frac{\partial u_j}{\partial t})] + S_M \end{aligned}$$

In the equations, $\rho$ indicates density, *u* indicates velocity vector, and the terms *P*, $u_{eff}$, and $S_M$ represent modified pressure, effective viscosity accounting for turbulence, and the sum of body forces, respectively. The turbulence model is solved by using the shear stress transport (SST) model, which operates by solving a turbulence/frequency-based model ($k-\omega$) at the wall and $k-\epsilon$ in the bulk flow [14, 38].

TAWSS is a key parameter for detecting the critical locations on AAA prone to rupture. The formulation of TAWSS is defined in Eq. 3. For calculating TAWSS, the absolute values of x, y, and z components of WSS are used within the time period of *T*. In the general approach, the time period (*T*) is used as one full cardiac cycle. In Eq. 3, $\tau _w$ defines the instantaneous WSS vector.

3 $$\begin{aligned} TAWSS = \frac{1}{T}\left( \int _0^T |\tau _w|dt\right) \end{aligned}$$

**Boundary conditions:** ANSYS CFX (ANSYS, Inc., Canonsburg, Pennsylvania, USA) was used to perform transient fluid flow simulations using a 0.01-time step for three seconds corresponding to three cardiac cycles. Three cardiac cycles were defined for the computational model in this study, and the third cycle was used in the analysis to eliminate the first cycle’s initial effects.

Previous research has shown that as blood flow from the heart enters the aorta, it is not fully developed and is not distributed symmetrically [5, 15–17, 19]. As a result, in this study, the input and output boundary conditions (Fig. 2a) were specified as normal to the surface boundary condition, and the same velocity and pressure profiles (Fig. 2b and c) were collected from the literature [39] used for all simulations. Each synthetic geometry’s CFD analysis required an average of 1 h, and the entire analysis consumed 230 h and 8 TB of space.

Transient CFD analysis yielded 300 WSS data (3 s/0.01 times step size=300 steps) for each condition. However, only the third cycle (1 s/0.01 times step size=100 steps) data were considered, ending up with 100 results exported for each geometry. Taking the average of all 100-time steps for each node resulted in a single CFD TAWSS profile for each analyzed geometry.

### Incorporating curvature with the geometry

**Fig. 3:**
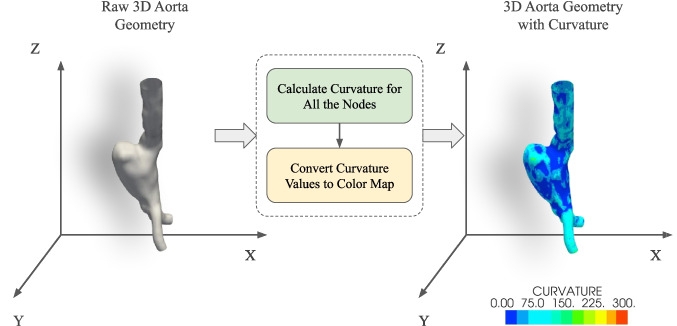
Raw aorta geometry (left). Aorta geometry with surface curvature represented through colormap (right)

The aorta geometries used in CFD simulations are referred to as the raw geometries. These geometries have no scalar values associated with them. When the input geometry is rendered, it looks similar to the geometry shown on the left side in Fig. 3. The shape and morphology of this geometry are represented by the shading created by the PyVista [40] software.

Curvature is a fundamental aspect for studying the geometric features of a surface. It quantifies the rate at which the tangent vector changes around a surface point. In other words, it is the amount by which the surface deviates from being a plane. Because of its ability to characterize the shape of a surface, it has been used as a crucial feature in several geometric DL problems [41–43]. A correlation has also been observed between the curvature of points on a blood vessel and WSS levels on these points as high curvature points were exposed to low WSS [44]. In our case, the curvature of the aortic surface can reflect the surface features like bulging, which is a crucial indicator of an aneurysm. On the other hand, we can detect an aneurysm from the TAWSS values of the aortic surface. We hypothesized that the surface curvature of the aortic geometry could be loosely correlated to the TAWSS values obtained from the CFD simulations. To test this hypothesis, we conducted experiments where we incorporated the surface curvature values of the aorta geometry with the 3D geometry itself as a color code, as shown in the right part of Fig. 3. Incorporating the curvature feature into the raw geometry improved the prediction capability of our DL model as presented in the Sect. 3.

### Geometry to image conversion

Instead of using 3D geometry descriptors such as voxel grid or polygon mesh, we utilized the rendered view of the geometry taken from different angles as the input to our hemodynamics prediction model. This approach is similar to the MultiView approach introduced in [45], where the authors showed that a 3D shape can be recognized by its rendered views from different angles. This was achieved with a classification accuracy far higher than other 3D descriptor-based techniques. Converting 3D geometries into rendered images has several advantages, such as conversion of non-Euclidean structured data of the 3D geometry into Euclidean representation is suitable for ANN and CNN models; conversion of the geometry into images significantly reduces its dimensionality, lowering the computational cost during analysis; and each image generated from the geometry can be treated as a separate training example, which increases the size of training data and helps reduce overfitting when training.

**Fig. 4:**
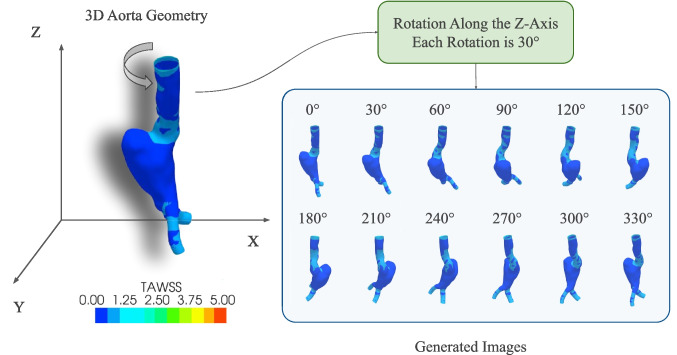
Generation of images from a rendered aorta geometry using the MultiView approach. Each image represents a particular rotation state around the *z*-axis

To convert the geometries into images, first, they were rendered using the PyVista [40] software. A Python script was used to rotate the geometry around the *z*-axis, and after each rotation step, a snapshot image of the rendered view was taken. In each step, the geometry was rotated $30^\circ$ counterclockwise. The captured images had a dimension of $768\times 768$ pixels. These images were downsampled to $256\times 256$ pixels and later used to train the DL model. The process of generating images from a 3D geometry is illustrated in Fig. 4.

### Data augmentation

By creating additional and distinct instances for training datasets, data augmentation helps DL models to perform better with more accurate results. A large and sufficient dataset is also required for better results. Several image augmentation techniques have been used extensively in image classification and segmentation problems. However, color-based augmentation techniques are not applicable in this work because they would change the values represented by the colormap. Also, image scaling-based augmentations cannot be used because they will distort the morphology of the geometry. With these facts under consideration, we applied rotation-based augmentation and random-zoom augmentation. The random-zoom augmentation was done at runtime when the model was training. However, the rotation-based augmentation was done beforehand while generating the image dataset.

**Fig. 5:**
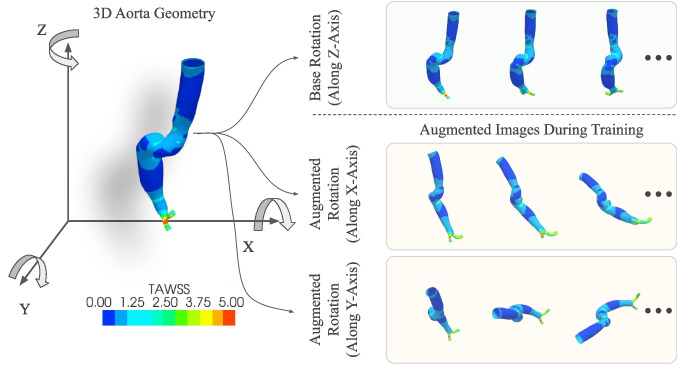
Axial rotation-based augmentation technique. The *z*-axis was the base axis of rotation to generate the original train and test set images. The *x*- and *y*-axes rotations were used to augment images into the training set

We utilized an axial rotation of the rendered geometry to augment additional images from the geometry. The *z*-axis was chosen as the base axis of rotation to generate the images using the MultiView approach discussed earlier. Rotation around the *x*- and *y*-axes was also introduced to augment more images into the training set. In other words, the training set contained images generated from all 3 axial rotations, while the test set only contained *z*-axis rotation-based images. The *x*- and *y*-axes rotation-based images were treated as augmented images while training our models. This process is demonstrated in Fig. 5.

**Fig. 6:**
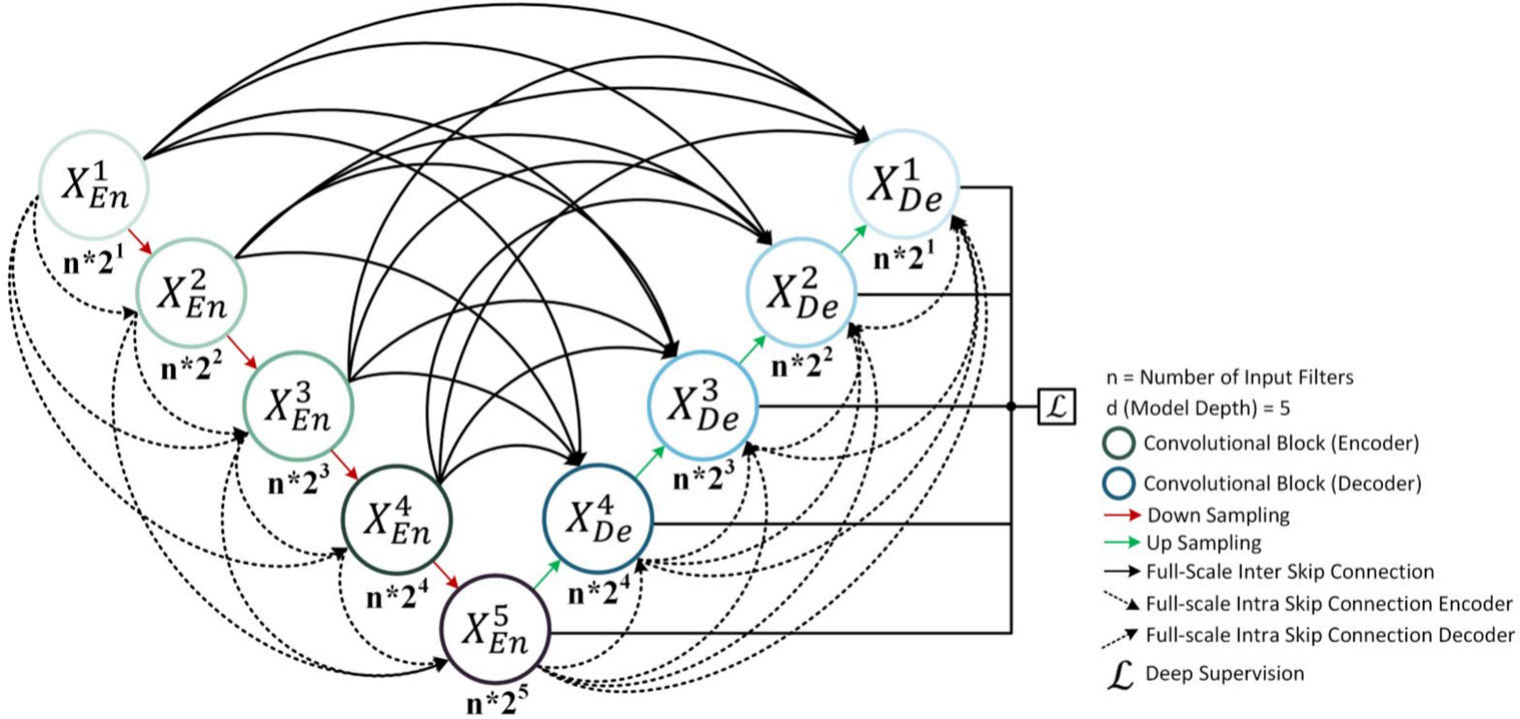
The architecture of the proposed MultiViewUNet network

### The proposed network architecture

In our previous discussion, we demonstrated the transformation of the geometry-to-geometry translation problem into an image-to-image translation task. This conversion allows us to leverage well-established deep learning architectures from the image processing domain, particularly focusing on state-of-the-art image segmentation networks that have been refined over the past decade.

The UNet architecture and its variants have demonstrated exceptional performance in image segmentation and image-to-image translation tasks [46, 47]. Our proposed MultiViewUNet combines the nested dense connectivity pattern of UNet++ with the full-scale skip connections of UNet3+ [48], while introducing comprehensive intra-skip connections in both encoder and decoder paths. Figure 6 illustrates our implementation of a 5-depth MultiViewUNet model.

#### Base architecture

The foundation of our network builds upon the original UNet [49], with significant enhancements to the skip connection patterns. Each convolutional block implements a sequence of two 3$\times$3 convolutional layers, each followed by batch normalization and ReLU activation. The spatial dimension reduction between encoder blocks is achieved through 2$\times$2 max-pooling operations with a stride of 2, while feature channels are doubled after each downsampling, starting from n initial filters.

#### Multi-scale feature aggregation

Our architecture implements a comprehensive feature aggregation strategy that operates at multiple scales through three distinct types of skip connections. The aggregation process is carefully designed to maintain both fine spatial details and rich semantic information throughout the network.

1. **Full-scale inter skip connections:** These connections facilitate direct feature transfer between encoder and decoder paths across different scales. For a given decoder block $X_{De}^d$, the inter-skip connections aggregate features from all encoder blocks through:

4 $$\begin{aligned} F_{inter}^d = Conv_{1\times 1}\Big (Concat\big [\{S(X_{En}^i, d)\}_{i=1}^{d}\big ]\Big ) \end{aligned}$$

where $S(X_{En}^i, d)$ represents the scaling operation (either max pooling or upsampling) necessary to match the spatial dimensions at depth *d*.

**Fig. 7:**
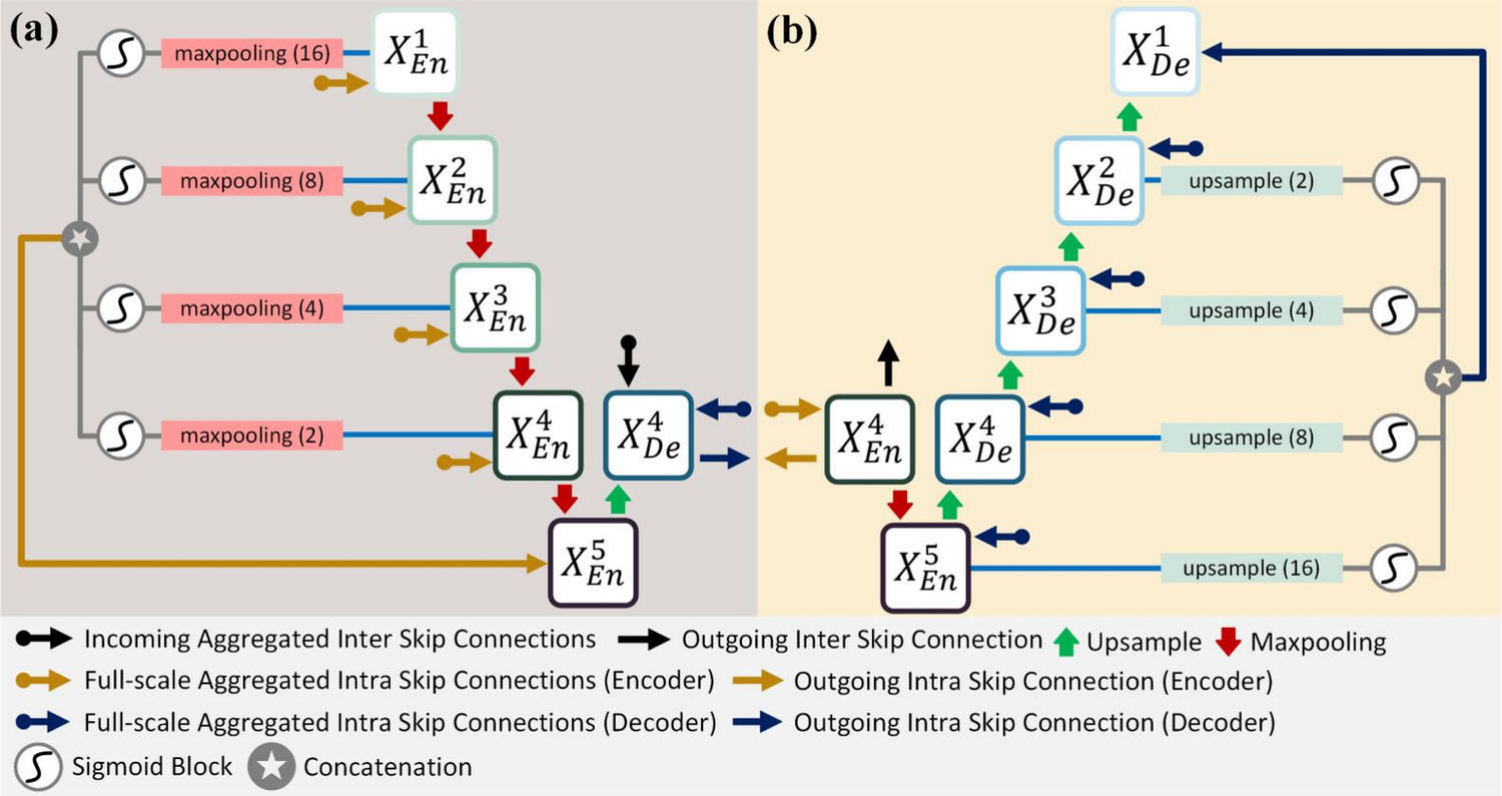
Full-scale aggregated feature map creation **a** for the fourth decoder layer $X^4_{De}$ and **b** from the fourth encoder Layer $X_{En}^4$

2. **Encoder intra-skip connections:** The encoder path implements a cascading feature aggregation scheme, illustrated in Fig. 7a. For an encoder block at depth *d*, the feature aggregation process is defined as:

5 $$\begin{aligned} X_{En}^d = Conv\Big (Concat\big [X_{En}^{d-1}, \{\sigma (MaxPool(X_{En}^i, 2^{d-i}))\}_{i=1}^{d-1}\big ]\Big ) \end{aligned}$$

In this formulation, $\sigma (\cdot )$ represents the sigmoid activation function, and $MaxPool(\cdot , k)$ denotes max pooling with factor *k*. Features from all previous layers are aggregated after appropriate scaling. This cascading structure creates a hierarchical feature representation where each level incorporates information from all previous scales. The sigmoid activation acts as a learned feature-gating mechanism, controlling the contribution of each scale to the final feature map.

3. **Decoder intra-skip connections:** The decoder path implements a complementary aggregation scheme, shown in Fig. 7b. For a decoder block at depth *d*, features are aggregated through:

6 $$\begin{aligned} X_{De}^d = Conv\Big (Concat\big [X_{De}^{d+1},\! \{\sigma (Up(X_{De}^i, 2^{i-d}))\}_{i=d+1}^D\big ]\Big ) \end{aligned}$$

Here, $Up(\cdot , k)$ represents bilinear upsampling by factor *k*, and *D* is the maximum network depth. Each upsampled feature map passes through a sigmoid activation to modulate its contribution to the final feature representation.

#### Feature aggregation implementation

The practical implementation of this feature aggregation system, as detailed in Fig. 7, involves several key components working in concert. The scale matching process is fundamental to the system’s operation, with the encoder path utilizing max pooling with factors of 16, 8, 4, and 2 for $X_{En}^1$ through $X_{En}^4$, while the decoder path employs bilinear upsampling with factors of 2, 4, 8, and 16 for progressive feature recovery.

Feature reweighting is accomplished through sigmoid blocks ($\mathcal {S}$) applied to each skip connection path. These blocks create adaptive feature gates that learn to emphasize relevant information, with the gating mechanism helping to manage the flow of information across different scales. The progressive aggregation system concatenates features at each level after appropriate scaling, with 1$\times$1 convolutions reducing the channel dimension after concatenation. This progressive nature ensures that each level has access to all relevant scales.

Figure 7 provides a detailed visualization of our feature aggregation process for the fourth layer. In Fig. 7a, we demonstrate the encoder-side intra-skip connections, where feature maps from previous layers are progressively aggregated through max-pooling operations. Figure 7b shows the decoder-side connections, where features are aggregated through upsampling operations. Both paths utilize sigmoid blocks for feature reweighting and concatenation for feature fusion. As shown in Fig. 7, the fourth decoder layer $X_{De}^4$ receives direct features from the corresponding encoder layer $X_{En}^4$, scaled features from all previous encoder layers through inter-skip connections, and progressive features from deeper decoder layers through intra-skip connections. This combination of aggregation paths enables the network to maintain both fine-grained spatial details from shallow layers and semantic information from deeper layers, while the sigmoid-based feature gating mechanism learns to optimally combine information across scales.

**Fig. 8:**
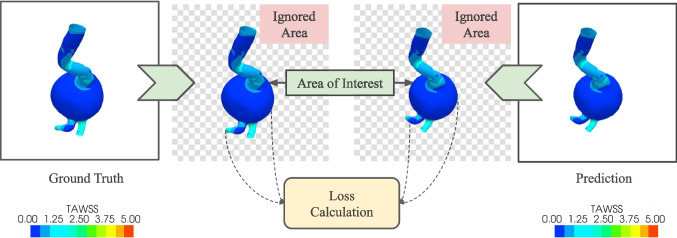
The effective loss calculation process. The loss is calculated over the area occupied by the geometry only. The surrounding while pixels are ignored during loss calculation

#### Implementation specifications

The network follows a carefully designed structure where the number of feature channels at depth *d* is given by $n \times 2^d$, where *n* is the initial number of filters. Throughout the network, we employ:

- 3$\times$3 convolutions with padding=1 for feature extraction
- 1$\times$1 convolutions for channel reduction in skip connections
- Sigmoid activation functions specifically for skip connection paths
- Batch normalization after each convolutional layer

This comprehensive skip connection system enables efficient information flow across all scales of the network. The combination of inter-skip connections for cross-path communication and intra-skip connections for within-path feature aggregation distinguishes our approach from previous UNet variants, leading to improved feature utilization while maintaining computational efficiency. More details about the model implementation can be found in “Appendix A: MultiViewUNet Architecture Details” in the Supplementary Materials.

### Optimization of loss function

Loss functions measure how far an estimated value is from its true value. In this case, the purpose of the loss function is to measure the difference between the estimated and target images for a particular geometry. The geometric structure remains the same for both predicted and target images. The only difference comes from the color of the images. Taking this into consideration, the mean squared error (MSE) was chosen as the loss function during the training process. MSE has been used extensively in image segmentation problems [50, 51], and it is also suitable in our case. MSE between the target and predicted values can be expressed by Eq. 7.

7 $$\begin{aligned} MSE = \frac{1}{N}\sum _{i=1}^N (Y(i) - \hat{Y}(i))^2 \end{aligned}$$

where *Y*(*i*) is the true intensity value at pixel *i*, $\hat{Y(i)}$ is the predicted intensity value at pixel *i*, and *N* is the total number of pixels in the image. This approach calculates the loss for all the pixels in the image. However, we are only interested in the pixels representing the geometric shape. The white pixels outside the shape do not convey any useful information thus, they can be ignored during loss calculation. Since the area in the image representing the geometry is the same for both input and target geometries, we can calculate the loss for only those pixels. So, we can redefine the loss function to calculate the effective MSE (EMSE) by the following equation.

8 $$\begin{aligned} EMSE = \frac{1}{N_e}\sum _{i_e=1}^{N_e} (Y(i_e) - \hat{Y}(i_e))^2 \end{aligned}$$

Here, $Y(i_e)$ is the true intensity value at the pixel $i_e$, $\hat{Y}(i_e)$ is the predicted intensity value at the pixel $i_e$, and $N_e$ is the total number of pixels representing the geometric shape. The effective loss calculation process is demonstrated in Fig. 8.

### Training and validation

We worked on a total of 23 original patient geometries and 230 synthetic geometries. Of the 230 synthetic geometries, 40 were taken into the test set, and the rest went to the train set. All of the 23 original patient geometries were placed into the test set. Therefore, the test set had a total of 63 geometries which is 25% of the total number of geometries. We ensured no data was leaking by creating the train and test sets before generating the images from them.

We used the Tensorflow [52] Python library to implement the proposed network. For the loss function, we used the EMSE loss discussed earlier. The loss was minimized using the Adam [53] optimizer with a learning rate of 0.0001. The network was trained for a maximum of 300 epochs with an early stopping criterion set on the validation loss with a tolerance of 30 epochs.

**Table 1:** Metrics for TWASS prediction from curvature of the aorta geometry

Model	NMAE	NMSE	NRMSE	EPSNR	Inference time
UNet [49]	0.414%	0.086%	1.305%	23.67 dB	8.02 ± 0.89 ms
Pix2Pix [54]	0.379%	0.032%	0.805%	27.3 dB	8.13 ± 0.91 ms
PointNet [55]	4.52%	0.573%	7.57%	18.3 dB	6.45 ± 0.61 ms
Proposed	0.362%	0.021%	0.643%	29.79 dB	11.85 ± 1.80 ms

### Evaluation metrics

Several evaluation metrics were used to evaluate the model’s performance quantitatively. Normalized mean absolute error (NMAE), normalized mean squared error (NMSE), normalized root mean squared error (NRMSE), and peak signal-to-noise ratio (PSNR) metrics have been used extensively in similar DL tasks. For our purpose, the effective values of the metrics were calculated by applying the same approach for loss calculation discussed earlier. The equations of these metrics are provided below.

9 $$\begin{aligned} EMAE= &   \frac{1}{N}\sum _{i_e=1}^{N_e} |Y(i_e) - \hat{Y}(i_e)|\end{aligned}$$

10 $$\begin{aligned} NMAE= &   \frac{EMAE}{max|S|}\end{aligned}$$

11 $$\begin{aligned} EMSE= &   \frac{1}{N_e}\sum _{i_e=1}^{N_e} (Y(i_e) - \hat{Y}(i_e))^2\end{aligned}$$

12 $$\begin{aligned} NMSE= &   \frac{EMSE}{max|S|}\end{aligned}$$

13 $$\begin{aligned} ERMSE= &   \sqrt{\frac{1}{N_e}\sum _{i_e=1}^{N_e} (Y(i_e) - \hat{Y}(i_e))^2}\end{aligned}$$

14 $$\begin{aligned} NRMSE= &   \frac{ERMSE}{max|S|}\end{aligned}$$

15 $$\begin{aligned} EPSNR= &   20 \cdot \log _{10}\left( \frac{MAX_I}{\sqrt{EMSE}}\right) \end{aligned}$$

Here, $Y(i_e)$ is the true intensity value at the pixel $i_e$, $\hat{Y}(i_e)$ is the predicted intensity value at the pixel $i_e$, $N_e$ is the total number of pixels representing the geometric shape, S is the max stress value obtained from CFD simulation, and $MAX_I$ is the maximum pixel intensity for the images.

## Results

In this section, we present the obtained experimental results where we tried to predict the TAWSS on the aortic wall from the aorta geometry as the input. As the ground truth of our experiments, we utilized the simulation results from CFD analysis.

### Results using the raw geometry rendering

First, we present the results we obtained from the experiment where only the raw geometry file rendering was used as the input to our pipeline. Since the raw geometry rendering does not have any associated features, the model relies entirely on the lighting and shading effect of the rendered images. We conducted several experiments where we tuned these effects to achieve the best combination. We utilized the physically based rendering (PBR) engine of the PyVista library and tuned the roughness and metallic properties of the renderings. We achieved the best results (NMAE=0.402%, NMSE=0.086%, NRMSE=1.316%, and EPSNR=23.67dB) with PBR turned on and “metallic” and “roughness” parameters set to 0.5.

Although the results are promising from this experiment, this approach has a drawback. The model only learns from shading of the rendered geometry, which depends on the surface of the geometry and lighting condition. The actual morphology of the surface is obstructed in this process. To improve the performance, we introduced the curvature feature of the surface and associated it with the raw geometry as colormap, as discussed in Sect. 2.3.

### Results using the curvature feature

In this section, we present the results we obtained from the experiment where we used the geometry with curvature values as the input to our pipeline. Since the curvature property reflects the morphology and shape of the geometry, the model could learn from these important parameters to predict the stress on the surface. As a result, the achieved metrics are much better than when we used only the raw geometry as the input.

**Fig. 9:**
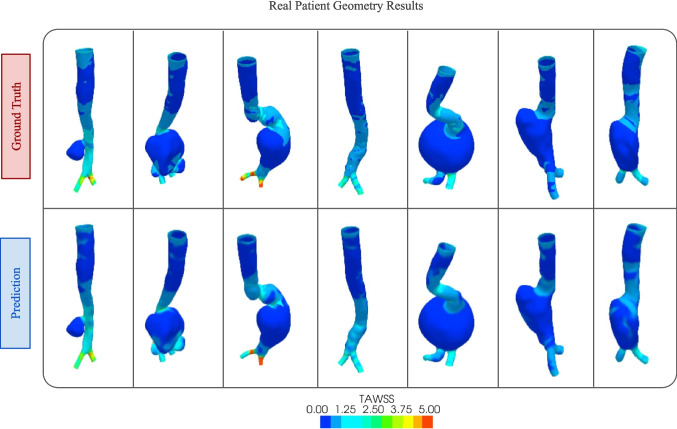
TAWSS prediction results on original patient geometries. The ground truth images from the CFD simulation are shown in the top row and the bottom row shows the predictions from our DL model

We have also compared the results of our proposed MultiViewUnet with several state-of-the-art networks. For the image-to-image domain we have chosen the UNet [49] and Pix2Pix [54] networks which have revolutionized image-to-image translation in recent years. We have also compared our results to a geometric approach called PointNet [55] which does not require images and instead uses the 3D geometries directly for DL analysis. PoinNet is one of the state-of-the-art networks for geometric deep learning. However, since the metrics are calculated directly from the 3D geometry instead of images, the metrics from this model are not fully comparable to our approach. Table 1 summarizes the comparison, where we can see that the proposed architecture performed significantly better than other networks with PixPix having the second-best performance. The inference time of our model is a little bit longer compared to other models due to its architectural complexity, but it is good enough for real-time applications. Also, it should be noted that the inference times have been measured on an NVIDIA T4 GPU.

Along with the quantitative results presented above, we have also reported some representative qualitative results below. Figure 9 shows the predictions from our model with their corresponding ground truth for some real patient geometries. We can see from the figure that the predicted results very closely resemble the simulation results obtained from CFD analysis.

**Fig. 10:**
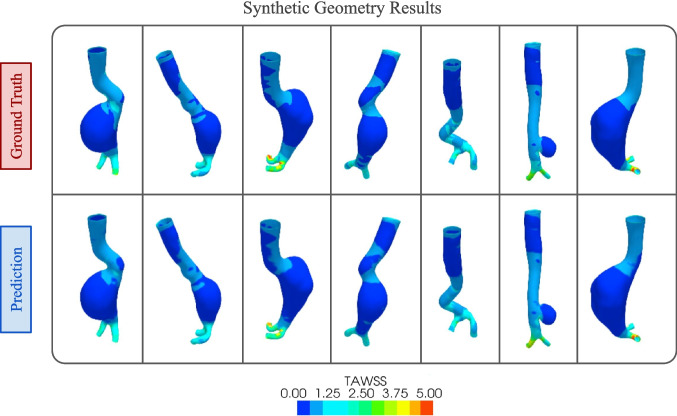
TAWSS prediction results on synthetic geometries. The ground truth images from the CFD simulation are shown in the top row and the bottom row shows the predictions from our DL model

Figure 10 shows the predictions from our model with their corresponding ground truth for some synthetic geometries. We can see from the figure that the predicted results very closely resemble the simulation results obtained from CFD analysis.

## Discussion

The use of DL algorithms in hemodynamics prediction is relatively new. To compare our results with similar investigations, in Table 2, some relevant works have been summarized. We reported on the works that focused on the aorta geometry specifically. From the table, we can see that all of the works either used CFD or finite element analysis (FEA) for their computational modeling. Most of the listed works focused on predicting hemodynamics parameters such as stress distribution, ECAP, or the velocity field with different ML algorithms except [32] where wall stresses such as Von Mises stresses were predicted.

**Table 2:** Comparison between literature studies for predicting hemodynamics using deep learning approaches in terms of computational modeling used to generate the target results to train DL models, the input and output of the DL model, deployed DL model, and the achieved results

Ref	Comp. model	Geometry	Output	Learning algorithm	Results
[32]	FEA	729 Thoracic Aorta Geometries	Aortic Wall Stress Distribution	PCA, MLP	Stress distribution: NMAE 0.492%, Peak stress value: NMAE 0.891%
[33]	CFD	300 LAA Geometries	ECAP	MLP, PCA, MLP	MLP: MAE 0.646% NMAE 4.720%, PCA, MLP: MAE 0.649% NMAE 5.756%
[57]	CFD	300 LAA geometries	ECAP	PCA, MLP, ED-CNN	PCA, MLP: MAE 0.73% ED-CNN: MAE 0.63%
[56]	CFD	729 Thoracic Aorta Geometries	Pressure Field, Velocity Field	MLP	Pressure Field: NMAE 1.427%, Velocity Field Magnitude: NMAE 1.961%
[58]	CFD	370 LAA Geometries	ECAP	PCA, MLP, ED-CNN, Geometric PointNet	PCA-MLP: MAE 0.608%, ED-CNN with Cartesian mapping input: MAE 0.651%, ED-CNN with Bull’s eye mapping input: MAE 0.654%, Geometric CNN: MAE 0.521%
Our	CFD	253 Abdominal Aorta Geometries	TAWSS	Multi-ViewUNet	Estimated stress distribution: NMAE 0.362%, NMSE 0.021%

**Fig. 11:**
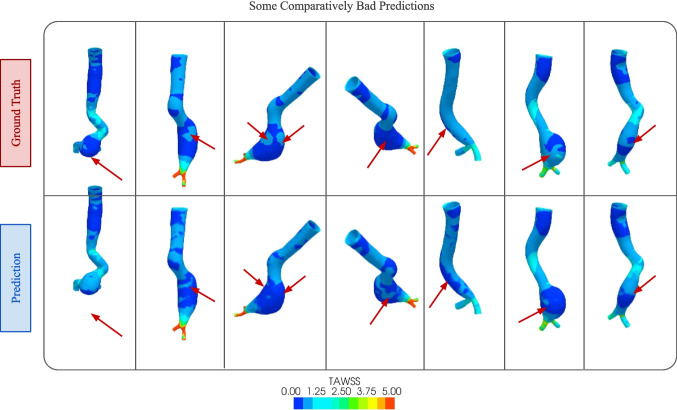
Some bad predictions from our proposed approach. The ground truth images from the CFD simulation are shown in the top row, and the bottom row shows the predictions from our DL model

The works done in [32, 56] utilized a dataset of 729 synthetic thoracic aorta geometries. Each geometry in this dataset contained exactly 5000 nodes which enabled the authors to use traditional ML and dimensionality reduction techniques like multilayer perceptron (MLP) and principal component analysis (PCA). [33, 57] also followed this approach. The authors of these works relied on an LAA dataset where each geometry contained exactly 2536 nodes. The fixed number of nodes is crucial for designing a common network architecture for all the geometries. However, in reality, patient-specific geometries are not uniform and may contain an arbitrary number of nodes. As a result, the methods proposed in these works are not directly applicable for other investigations. Our proposed approach on the other hand does not have this limitation and can be applied to any geometric shape regardless of the mesh type and the number of nodes. [58] uses a similar approach to ours that relies on a uniform number of geometry nodes. The authors also proposed a technique for flattening the geometric shape into a rectangular image using non-linear angular mapping. However, the generated image has no information about the heterogeneous anatomy of the aorta which was mentioned as a limitation by the authors. Our approach, however, retains the morphology of the aorta in the generated images and thus does not have this limitation.

Most of the work in this area reported the NMAE values to quantitatively access their predicted outputs. In these works, the NMAE metric was generated for the 3D geometry, where the error values were calculated over all the nodes and then averaged. Our work, however, primarily focuses on qualitative results as opposed to quantitative metrics because we are not producing the 3D geometry as the output of our model. As a result, we could not follow the exact approach to calculating the errors as done in the above-mentioned works. Instead, we used an indirect approach where we calculated the metrics from the output images which represent the geometry. In other words, the error was calculated from the difference in image pixel intensity values in the target and predicted images for a particular geometry. Due to this, our reported metrics may not be fully comparable to the other works, but we provided them for a general comparison.

Although the predictions from our algorithm are usually very good and closely resemble the CFD results, in a few cases, it produces less accurate results. Some of these predictions are shown in Fig. 11. The areas where our predictions did not match with the ground truth have been marked with red arrows. These types of discrepancies usually occur when stress is disturbed, and flow is turbulent, chaotic, and unpredictable. In straight aortic segments where flow is laminar, less deviation was observed. The main reason behind this inaccuracy may be related to our small dataset. Our dataset only contains 253 geometries which is a relatively small number for a DL analysis. Small datasets like ours are very prone to the overfitting issue. We introduced data augmentation and dropout to reduce overfitting. However, our predictions would have been more accurate if we had a larger dataset with more geometric variations.

One limitation of our approach is we converted the 3D geometries into a set of images, which were processed using an image-to-image generation neural network. In other words, the output of our proposed model is also a set of images representing the 3D geometry. While the images provide the stress values represented through color mapping and are sufficient for qualitative assessment, regenerating the 3D geometric representation from the images can be challenging.

Another drawback of the proposed approach is that we had to downscale the images to 256$\times$256 pixels due to computational limitations. As a result, the output images from the model are relatively low resolution and sometimes appear pixelated around the edges. Increasing the image resolution further requires a significant amount of RAM and GPU storage in the training phase, however, this can be fixed with good computational resources. In the future, we look forward to enhancing the output image resolution through another neural network and improving its quality.

## Conclusion and future works

We created a DL surrogate to replace time-consuming and computationally expensive CFD simulations for hemodynamics prediction. Our proposed algorithm was developed by experimenting on both real and synthetic aorta geometries with or without an aneurysm. In the process, a novel analysis technique was also developed for doing a geometry-to-geometry analysis with DL. The proposed MultiViewUNet approach simplified the way of using 3D geometric shapes as input and output of a neural network architecture by converting them into a set of images. With a relatively mature image-to-image domain in the DL world, many state-of-the-art NN architectures can be experimented with for this and related tasks. In this work, we achieved a good NMAE score of 0.362% for aortic TAWSS prediction, and our qualitative results are also promising. Besides TAWSS, we look forward to predicting other indicators of the aneurysm, such as ECAP, OSI, and RRT, using our approach in the future. We also plan on exploring generative algorithms (GAN) in the future instead of UNet-based architectures. We believe that our proposed method will enable real-time analysis of hemodynamics and organ stress analysis for several clinical applications.

## Supplementary Information

Below is the link to the electronic supplementary material.

Supplementary file 1 (pdf 138 KB)

Supplementary file 2 (tex 4 KB)

## Data Availability

The data used in this study can be made available upon a reasonable request to the corresponding author.

## Code availability

The source code for this article can be found on GitHub: https://github.com/atick-faisal/MultiViewUNet-Aneurysm.

## References

[1] McAloon C, Osman F, Glennon P, Lim P, Hayat S (2016) Global epidemiology and incidence of cardiovascular disease 57–96

[2] McGloughlin TM, Doyle BJ (2010) New approaches to abdominal aortic aneurysm rupture risk assessment: engineering insights with clinical gain. Arterioscler Thromb Vasc Biol 30(9):1687–1694 20508202 10.1161/ATVBAHA.110.204529

[3] Sakalihasan N, Limet R, Defawe OD (2005) Abdominal aortic aneurysm. The Lancet 365(9470):1577–1589 10.1016/S0140-6736(05)66459-8 15866312

[4] Kontopodis N, Metaxa E, Papaharilaou Y, Tavlas E, Tsetis D, Ioannou C (2015) Advancements in identifying biomechanical determinants for abdominal aortic aneurysm rupture. Vascular 23(1):65–77 24757027 10.1177/1708538114532084

[5] Salman HE, Ramazanli B, Yavuz MM, Yalcin HC (2019) Biomechanical investigation of disturbed hemodynamics-induced tissue degeneration in abdominal aortic aneurysms using computational and experimental techniques. Front Bioeng Biotechnol 7:111 31214581 10.3389/fbioe.2019.00111 PMC6555197

[6] Salman H, Yalcin H (2020) Computational investigation of the effect of wall thickness on rupture risk in abdominal aortic aneurysms. J Appl Fluid Mech 14(2):499–513

[7] Di Martino E, Mantero S, Inzoli F, Melissano G, Astore D, Chiesa R, Fumero R (1998) Biomechanics of abdominal aortic aneurysm in the presence of endoluminal thrombus: experimental characterisation and structural static computational analysis. Eur J Vasc Endovasc Surg 15(4):290–299 9610340 10.1016/s1078-5884(98)80031-2

[8] Di Martino ES, Vorp DA (2003) Effect of variation in intraluminal thrombus constitutive properties on abdominal aortic aneurysm wall stress. Annals Biomed Eng 31:804–809 10.1114/1.1581880 12971613

[9] Di Martino ES, Guadagni G, Fumero A, Ballerini G, Spirito R, Biglioli P, Redaelli A (2001) Fluid–structure interaction within realistic three-dimensional models of the aneurysmatic aorta as a guidance to assess the risk of rupture of the aneurysm. Med Eng Phys 23(9):647–655 11755809 10.1016/s1350-4533(01)00093-5

[10] Di Martino ES, Bohra A, Geest JPV, Gupta N, Makaroun MS, Vorp DA (2006) Biomechanical properties of ruptured versus electively repaired abdominal aortic aneurysm wall tissue. J Vasc Surg 43(3):570–576 16520175 10.1016/j.jvs.2005.10.072

[11] Hasan M, Al-Thani H, El-Menyar A, Zeidan A, Al-Thani A, Yalcin HC (2023) Disturbed hemodynamics and oxidative stress interaction in endothelial dysfunction and AAA progression: focus on Nrf2 pathway. Int J Cardiol 389 10.1016/j.ijcard.2023.131238 37536420

[12] Al-Thani H, Yalcin H, Mutlu O, El-Menyar A (2023) Hemodynamics analysis and computational fluid dynamics simulations to identify potential location of rupture in abdominal aortic aneurysm. Eur Heart J 44(Supplement_2):655–2019

[13] Mutlu O, Salman HE, Al-Thani H, El-Menyar A, Qidwai UA, Yalcin HC (2023) How does hemodynamics affect rupture tissue mechanics in abdominal aortic aneurysm: focus on wall shear stress derived parameters, time-averaged wall shear stress, oscillatory shear index, endothelial cell activation potential, and relative residence time. Comput Biol Med 154 10.1016/j.compbiomed.2023.106609 36724610

[14] Menter F (1993) Zonal two equation kw turbulence models for aerodynamic flows. In: 23rd Fluid dynamics, plasmadynamics, and lasers conference, p 2906

[15] Weigang E, Kari FA, Beyersdorf F, Luehr M, Etz CD, Frydrychowicz A, Harloff A, Markl M (2008) Flow-sensitive four-dimensional magnetic resonance imaging: flow patterns in ascending aortic aneurysms. Eur J Cardiothorac Surg 34(1):11–16 18515137 10.1016/j.ejcts.2008.03.047

[16] Ha H, Kim GB, Kweon J, Lee SJ, Kim Y-H, Kim N, Yang DH (2016) The influence of the aortic valve angle on the hemodynamic features of the thoracic aorta. Sci Reports 6(1):32316 10.1038/srep32316 PMC4999809 27561388

[17] Mutlu O, Salman HE, Yalcin HC, Olcay AB (2021) Fluid flow characteristics of healthy and calcified aortic valves using three-dimensional Lagrangian coherent structures analysis. Fluids 6(6):203

[18] Salman HE, Saltik L, Yalcin HC (2021) Computational analysis of wall shear stress patterns on calcified and bicuspid aortic valves: focus on radial and coaptation patterns. Fluids 6(8):287

[19] Olcay A, Amindari A, Kirkkopru K, Yalcin H (2018) Characterization of disturbed hemodynamics due to stenosed aortic jets with a Lagrangian coherent structures technique

[20] Xenos M, Rambhia SH, Alemu Y, Einav S, Labropoulos N, Tassiopoulos A, Ricotta JJ, Bluestein D (2010) Patient-based abdominal aortic aneurysm rupture risk prediction with fluid structure interaction modeling. Ann Biomed Eng 38:3323–3337 10.1007/s10439-010-0094-3 20552276

[21] Qiu Y, Yuan D, Wen J, Fan Y, Zheng T (2018) Numerical identification of the rupture locations in patient-specific abdominal aortic aneurysmsusing hemodynamic parameters. Comput Methods Biomech Biomed Eng 21(1):1–12 10.1080/10255842.2017.1410796 29251991

[22] Fillinger MF, Raghavan ML, Marra SP, Cronenwett JL, Kennedy FE (2002) In vivo analysis of mechanical wall stress and abdominal aortic aneurysm rupture risk. J Vasc Surg 36(3):589–597 12218986 10.1067/mva.2002.125478

[23] Wang X, Li X (2013) A fluid-structure interaction-based numerical investigation on the evolution of stress, strength and rupture potential of an abdominal aortic aneurysm. Comput Methods Biomech Biomed Eng 16(9):1032–1039 10.1080/10255842.2011.652097 22289116

[24] Venkatasubramaniam A, Fagan M, Mehta T, Mylankal K, Ray B, Kuhan G, Chetter I, McCollum P (2004) A comparative study of aortic wall stress using finite element analysis for ruptured and non-ruptured abdominal aortic aneurysms. Eur J Vasc Endovasc Surg 28(2):168–176 15234698 10.1016/j.ejvs.2004.03.029

[25] Chen C (2024) Ascent of machine learning in medicine 10.1038/s41563-019-0360-1 31000807

[26] Aggarwal S, Pandey K (2023) Early identification of PCOS with commonly known diseases: obesity, diabetes, high blood pressure and heart disease using machine learning techniques. Expert Syst Appl 217

[27] Hossain ME, Uddin S, Khan A (2021) Network analytics and machine learning for predictive risk modelling of cardiovascular disease in patients with type 2 diabetes. Expert Syst Appl 164:113918

[28] Brunton SL, Noack BR, Koumoutsakos P (2020) Machine learning for fluid mechanics. Ann Rev Fluid Mech 52:477–508

[29] Kutz JN (2017) Deep learning in fluid dynamics. J Fluid Mech 814:1–4

[30] Li G, Sun H, He J, Ding X, Zhu W, Qin C, Zhang X, Zhou X, Yang B, Guo Y (2024) Deep learning, numerical, and experimental methods to reveal hydrodynamics performance and cavitation development in centrifugal pump. Expert Syst Appl 237:121604

[31] Lopez-Martin M, Le Clainche S, Carro B (2021) Model-free short-term fluid dynamics estimator with a deep 3d-convolutional neural network. Expert Syst Appl 177:114924

[32] Liang L, Liu M, Martin C, Sun W (2018) A deep learning approach to estimate stress distribution: a fast and accurate surrogate of finite-element analysis. J R Soc Interface 15(138):20170844 29367242 10.1098/rsif.2017.0844 PMC5805990

[33] Morales X, Mill J, Juhl KA, Olivares A, Jimenez-Perez G, Paulsen RR, Camara O (2020) Deep learning surrogate of computational fluid dynamics for thrombus formation risk in the left atrial appendage. In: Statistical atlases and computational models of the heart. Multi-Sequence CMR Segmentation, CRT-EPiggy and LV Full Quantification Challenges: 10th International Workshop, STACOM 2019, Held in Conjunction with MICCAI 2019, Shenzhen, China, October 13, 2019, Revised Selected Papers 10, pp 157–166. Springer

[34] Rawat W, Wang Z (2017) Deep convolutional neural networks for image classification: a comprehensive review. Neural Comput 29(9):2352–2449 28599112 10.1162/NECO_a_00990

[35] Silva LC, Sobrinho ÁAdCC, Cordeiro TD, Melo RF, Bittencourt II, Marques LB, Cunha Matos DDM, Silva AP, Isotani S (2023) Applications of convolutional neural networks in education: a systematic literature review. Expert Syst Appl 120621

[36] Monti F, Boscaini D, Masci J, Rodola E, Svoboda J, Bronstein MM (2017) Geometric deep learning on graphs and manifolds using mixture model CNNs. In: Proceedings of the IEEE conference on computer vision and pattern recognition, pp 5115–5124

[37] Wang Y, Sun Y, Liu Z, Sarma SE, Bronstein MM, Solomon JM (2019) Dynamic graph CNN for learning on point clouds. ACM Trans Graph (tog) 38(5):1–12

[38] Bardina J, Huang P, Coakley T, Bardina J, Huang P, Coakley T (1997) Turbulence modeling validation. In: 28th Fluid dynamics conference, p 2121

[39] Gao Z, Xiong J, Chen Z, Deng X, Xu Z, Sun A, Fan Y (2020) Gender differences of morphological and hemodynamic characteristics of abdominal aortic aneurysm. Biol Sex Differ 11:1–10 32693818 10.1186/s13293-020-00318-3 PMC7372899

[40] Sullivan CB, Kaszynski A (2019) PyVista: 3d plotting and mesh analysis through a streamlined interface for the visualization toolkit (VTK). J Open Source Softw 4(37):1450 10.21105/joss.01450

[41] He W, Jiang Z, Zhang C, Sainju AM (2020) Curvanet: geometric deep learning based on directional curvature for 3d shape analysis. In: Proceedings of the 26th ACM SIGKDD international conference on knowledge discovery & data mining, pp 2214–2224

[42] Bongratz F, Rickmann A-M, Pölsterl S, Wachinger C (2022) Vox2cortex: fast explicit reconstruction of cortical surfaces from 3d MRI scans with geometric deep neural networks. In: Proceedings of the IEEE/CVF conference on computer vision and pattern recognition, pp 20773–20783

[43] Cheng H, Zhu J, Lu J, Han X (2024) Edgcnet: joint dynamic hyperbolic graph convolution and dual squeeze-and-attention for 3d point cloud segmentation. Expert Syst Appl 237

[44] Wahle A, Lopez JJ, Olszewski ME, Vigmostad SC, Chandran KB, Rossen JD, Sonka M (2006) Plaque development, vessel curvature, and wall shear stress in coronary arteries assessed by x-ray angiography and intravascular ultrasound. Med Image Anal 10(4):615–631 16644262 10.1016/j.media.2006.03.002 PMC2590653

[45] Su H, Maji S, Kalogerakis E, Learned-Miller E (2015) Multi-view convolutional neural networks for 3d shape recognition. In: Proceedings of the IEEE international conference on computer vision, pp 945–953

[46] Cheng H-D, Jiang XH, Sun Y, Wang J (2001) Color image segmentation: advances and prospects. Pattern Recognit 34(12):2259–2281

[47] Minaee S, Boykov Y, Porikli F, Plaza A, Kehtarnavaz N, Terzopoulos D (2021) Image segmentation using deep learning: a survey. IEEE Trans Pattern Anal Mach Intell 44(7):3523–3542 10.1109/TPAMI.2021.3059968 33596172

[48] Huang H, Lin L, Tong R, Hu H, Zhang Q, Iwamoto Y, Han X, Chen Y-W, Wu J (2020) Unet 3+: a full-scale connected unet for medical image segmentation. In: ICASSP 2020-2020 IEEE international conference on acoustics, speech and signal processing (ICASSP), pp 1055–1059. IEEE

[49] Ronneberger O, Fischer P, Brox T (2015) U-net: convolutional networks for biomedical image segmentation. In: Medical Image Computing and Computer-assisted intervention–MICCAI 2015: 18th International Conference, Munich, Germany, October 5-9, 2015, Proceedings, Part III 18, pp 234–241. Springer

[50] Chen C, Qin C, Qiu H, Tarroni G, Duan J, Bai W, Rueckert D (2020) Deep learning for cardiac image segmentation: a review. Front Cardiovasc Med 7:25 32195270 10.3389/fcvm.2020.00025 PMC7066212

[51] Haque IRI, Neubert J (2020) Deep learning approaches to biomedical image segmentation. Inform Med Unlocked 18:100297

[52] Abadi M, Agarwal A, Barham P, Brevdo E, Chen Z, Citro C, Corrado GS, Davis A, Dean J, Devin M, Ghemawat S, Goodfellow I, Harp A, Irving G, Isard M, Jia Y, Jozefowicz R, Kaiser L, Kudlur M, Levenberg J, Mané D, Monga R, Moore S, Murray D, Olah C, Schuster M, Shlens J, Steiner B, Sutskever I, Talwar K, Tucker P, Vanhoucke V, Vasudevan V, Viégas F, Vinyals O, Warden P, Wattenberg M, Wicke M, Yu Y, Zheng X (2015) TensorFlow: large-scale machine learning on heterogeneous systems. Software available from tensorflow.org. https://www.tensorflow.org/

[53] Kingma DP, Ba J (2014) Adam: a method for stochastic optimization. arXiv preprint arXiv:1412.6980

[54] Isola P, Zhu J-Y, Zhou T, Efros AA (2017) Image-to-image translation with conditional adversarial networks. In: Proceedings of the IEEE conference on computer vision and pattern recognition, pp 1125–1134

[55] Qi CR, Su H, Mo K, Guibas LJ (2017) Pointnet: deep learning on point sets for 3d classification and segmentation. In: Proceedings of the IEEE conference on computer vision and pattern recognition, pp 652–660

[56] Liang L, Mao W, Sun W (2020) A feasibility study of deep learning for predicting hemodynamics of human thoracic aorta. J Biomech 99:109544 31806261 10.1016/j.jbiomech.2019.109544

[57] Acebes C, Morales X, Camara O (2021) A cartesian grid representation of left atrial appendages for a deep learning estimation of thrombogenic risk predictors. In: Statistical atlases and computational models of the heart. M &Ms and EMIDEC Challenges: 11th International Workshop, STACOM 2020, Held in Conjunction with MICCAI 2020, Lima, Peru, October 4, 2020, Revised Selected Papers 11, pp 35–43. Springer

[58] Morales Ferez X, Mill J, Juhl KA, Acebes C, Iriart X, Legghe B, Cochet H, De Backer O, Paulsen RR, Camara O (2021) Deep learning framework for real-time estimation of in-silico thrombotic risk indices in the left atrial appendage. Front Psychol 12:694945 10.3389/fphys.2021.694945 PMC8274486 34262482

