# Supplementary material for: Rapid wall shear stress prediction for aortic aneurysms using deep learning: a fast alternative to CFD
Source: Med Biol Eng Comput. 2025 Feb 17;63(7):2173–90. doi: 10.1007/s11517-025-03311-3 (PMC12204922; doi:10.1007/s11517-025-03311-3)
Supplement: Supplementary file 1 — (pdf 138 KB) [file 11517_2025_3311_MOESM1_ESM.pdf]

Appendix A: MultiViewUNet Architecture Details

A.1 Network Architecture

The MultiViewUNet architecture maps an input image $\mathbf{X} \in \mathbb{R}^{H \times W \times C}$ to an output $\mathbf{Y} \in \mathbb{R}^{H \times W \times C}$, where $H = W = 256$ and $C = 3$ in our implementation. The network combines nested dense connectivity patterns with comprehensive full-scale skip connections in both encoder and decoder paths.

A.2 Architectural Components

A.2.1 Base Network Structure

The network consists of five depth levels ($d = 1, \ldots, 5$), where each level contains encoder blocks X_{En}^d and decoder blocks X_{De}^d. The number of feature channels at depth d is given by:

$$n_d = n \times 2^d \tag{1}$$

where n is the initial number of input filters.

A.2.2 Basic Block Structure

Each convolutional block implements:

$$F_{block}(\mathbf{X}) = \text{Conv}_{3\times3}(\text{BN}(\text{ReLU}(\text{Conv}_{3\times3}(\text{BN}(\text{ReLU}(\mathbf{X})))))) \tag{2}$$

where $\text{Conv}_{3\times3}$ denotes a convolutional layer with kernel size 3×3 and padding 1, BN represents batch normalization, and ReLU is the rectified linear unit activation.

A.3 Feature Aggregation Components

A.3.1 Full-Scale Inter Skip Connections

For a decoder block at depth d, the inter-skip connection aggregation is defined as:

$$F_{inter}^d = \text{Conv}_{1\times1}\Big(\text{Concat}\big[\{S(X_{En}^i, d)\}_{i=1}^d\big]\Big) \tag{3}$$

where $S(X_{En}^i, d)$ represents the appropriate scaling operation to match the spatial dimensions of depth d.

A.3.2 Encoder Intra-Skip Connections

The encoder path implements cascading connections defined by:

$$X_{En}^d = \text{Conv}\Big(\text{Concat}\big[X_{En}^{d-1}, \{\sigma(\text{MaxPool}(X_{En}^i, 2^{d-i}))\}_{i=1}^{d-1}\big]\Big) \tag{4}$$

The scaling factors for max pooling operations are:

$$k_{pool}^d = 2^{d-1}, \quad d = 1, \ldots, 5 \tag{5}$$

A.3.3 Decoder Intra-Skip Connections

The decoder path implements upsampling connections defined by:

$$X_{De}^{d} = \text{Conv}\Big(\text{Concat}\big[X_{De}^{d+1}, \{\sigma(\text{Up}(X_{De}^{i}, 2^{i-d}))\}_{i=d+1}^{D}\big]\Big) \tag{6}$$

The upsampling factors are given by:

$$k_{up}^{d} = 2^{d-1}, \quad d = 1, \ldots, 5 \tag{7}$$

A.4 Implementation Details

A.4.1 Scale Matching Operations

The network implements two types of scaling operations:

$$\text{MaxPool}(\mathbf{X}, k) = \text{MaxPool}_{k\times k}(\mathbf{X}) \tag{8}$$

$$\text{Up}(\mathbf{X}, k) = \text{BilinearUpsampling}_{k\times k}(\mathbf{X}) \tag{9}$$

A.4.2 Feature Reweighting

The sigmoid-based feature gating mechanism is defined as:

$$\sigma(\mathbf{X}) = \frac{1}{1 + e^{-\mathbf{X}}} \tag{10}$$

A.4.3 Channel Reduction

After feature concatenation, channel reduction is performed through:

$$F_{reduce}(\mathbf{X}) = \text{Conv}_{1\times 1}(\mathbf{X}) \tag{11}$$

A.5 Training Configuration

The network is trained using the Mean Squared Error (MSE) loss function, defined as:

$$\mathcal{L}_{EMSE}(\mathbf{Y}, \hat{\mathbf{Y}}) = \frac{1}{N}\sum_{i=1}^{N_e}(\mathbf{Y}_i - \hat{\mathbf{Y}}_i)^2 \tag{12}$$

where $\mathbf{Y}$ represents the ground truth output, $\hat{\mathbf{Y}}$ is the network prediction, and N is the total number of elements in the output tensor.

The optimizer parameters are set to:

$$\begin{aligned} \text{Learning Rate} &= 1 \times 10^{-4} \\ \text{Adam} : \beta_1 = 0.9&, \beta_2 = 0.999 \end{aligned} \tag{13}$$
